# Endothelioma of Bone Following Injury1Read at the forty-first annual meeting of the Medical Association of Central New York, held at Buffalo. October 27, 1908.

**Published:** 1909-04

**Authors:** G. W. Cottis

**Affiliations:** Batavia, N. Y.


### Endothelioma of Bone Following Injury[1]

By G. W. COTTIS, M. D., Batavia, N. Y.

IT IS not my intention to discuss the oncologic position of endotheliomata or to go into the theories of their origin. It is sufficient in the way of definition to remind you that since all endothelium is of mesoblastic origin malignant tumors of this tissue are properly classed generically as sarcomata. At the same time their peculiarities of growth, as well as their sharply defined histogenesis, entitle them to be classed as a specifically distinct form of tumor. They have been considered to be intermediate between connective tissue tumors on the one hand and epithelial tumors on the other, and histologically we find that they present all transitions from diffuse sarcoma to adenocarcinoma, the diagnosis from the study of a given microscopic section being sometimes impossible.

Endothelioma of bone is a rare condition, if the number of recorded cases be accepted as a criterion. It is probable, however, that many cases are not differentiated from sarcoma or carcinoma. In 1905, Howard and Crile collected nineteen cases of

---

1. Read at the forty-first annual meeting of the Medical Association of Central New York, held at Buffalo. October 27, 1908.

endothelioma and perithelioma of bone, and added four cases of their own, making twenty-three in all. I have been able to find only one other case in the literature. This was reported by Dr. John A. Hartwell in *Annals of Surgery,* December, 1904, and overlooked by Howard and Crile. No cases have been reported since. The case on which the present paper is based is therefore the twenty-fifth to be recorded.

The ordinary textbooks of surgery do not mention this condition, and it is the desire to emphasise certain features which may aid in the diagnosis of a condition always serious and often obscure, that leads me to take up the time of this meeting with the report of a case.

The patient, male, aged 58, bookkeeper, was injured by stepping through an opening between a veranda and a flight of steps. He fell, striking his scrotum and right thigh against the edge of the flooring. The blow caused severe pain, with swelling and ecchymosis in the scrotum and hip. He was able to walk unassisted, and the next day went to work as usual. For three weeks he worked regularly, although each step caused lancinating pains in the hip and thigh. At the end of this time he consulted me.

Examination showed the remains of ecchymosis on the inner side of right thigh. There was spasm of the muscles of the thigh most marked in those of the adductor group, which were strongly contracted, holding the thigh in slight flexion. Pressure on, and to the inner side of Scarpa's triangle and also on the greater trochanter caused intense pain, as did passive motion of the hip joint. The patient walked with difficulty and pain, the latter becoming very severe when his toe happened to catch on the edge of a rug or other obstacle. Radiograms of the hip joint were made, and showed a faint line crossing the upper third of the head of the femur. A competent radiographer interpreted this as a fracture.

The patient was put to bed and the hip was immobilised in flexion by a plaster of Paris spica. This gave slight temporary relief from pain, but at the end of a week it was necessary to remove the spica. Extension with a 10 lb. weight was then tried, but this seemed to increase pain, and was discontinued. After being in bed two weeks—five weeks from the initial injury—the patient had complete anorexia, constipation, and an annoying glossitis. He was losing strength and flesh rapidly and was too weak to rise without assistance. Large purpuric spots appeared on the thighs and abdomen. There was at no time any rise in temperature.

Two months after the injury the inguinal and femoral lymph nodes became enlarged and tender. The most careful examination revealed nothing but extreme tenderness about the hip joint. There was no swelling, fluctuation or crepitus. Pains began to appear in other parts of the body, chiefly in the ribs, both anteriorly and posteriorly, in the scapulæ and humeri Emaciation was progressive. The skin was dry and harsh and slightly yellow. Glossitis became so severe as to almost prevent speech and it was difficult to feed the patient even on liquids.

About four months after the injury a blood examination was made. The findings, with the exception of the color index, at first suggested pernicious anemia. Hb., 50 per cent.; red cells, 2,600,000; leucocytis, 4,000.

The red cells showed large and small forms, poikilocytosis, polychromatophilia, and many nucleated cells, both normoblasts and megaloblasts. The differential leucocyte count showed: polynuclears, 27.6 per cent.; lymphocytes, 41.3 per cent.; large mononuclears, 6.6 per cent.; transitional (marrow cells) 6.3 per cent.; eosinophiles, 16.6 per cent.; mast cells, 1.3 per cent.; myelocytes, none. Of the nucleated red cells, 20 normoblasts were seen to 7 megaloblasts.

The differential count was confirmed for me by Dr. M. Warren, of the pathological laboratory of Cornell University. Dr. James Ewing, after looking over the slides and the history of the case expressed the opinion that it was not a primary pernicious anemia, but a blood condition due to bone marrow injury following trauma—possibly impacted fracture of the femur—or to multiple myeloma. The eosinophilia suggested intestinal parasites, but frequent examinations of the feces for ova were negative. A tentative diagnosis of multiple myeloma was made. Repeated urinary analyses, however, showed an entire absence of Bence-Jones proteid (albumosuria) which is always found with multiple myeloma. This left us with the diagnosis of malignant degeneration of the bone marrow, the exact nature of the change being undeterminable.

The ordinary treatment for pernicious anemia gave no results clinically, although the blood picture changed somewhat. Pain and tenderness in most of the bones of the trunk and limbs became so intense that the patient could be moved in bed only by lifting him on a sheet, held by the edges. He died of exhaustion, October 2, a little less than eight months from the date of his initial injury. During the entire course of the disease, pain had been constant and severe.

Autopsy revealed no gross lesions of the viscera, except extreme thinning of the stomach and intestines, and intense anemia. The inguinal lymph nodes, and those along the course of the external iliac artery, were enlarged, but not adherent.

The right hip joint was normal and showed no evidence of injury. The tissues about the neck of the femur were edematous, and at the level of the lesser trochanter was a small amount of disorganised tissue of gelatinous consistency. The shaft of the femur at this point was soft and of a greyish red color.

The softening extended vertically about half an inch and involved one half the circumference of the bone, and all of the central portion. The other half of the circumference consisted of a thin shell of bone and periosteum. There was no enlargement and no new growth of bone.

Microscopic examinations were made of the kidneys, liver, spleen, tongue, intestine, adrenal, sympathetic ganglia, mesenteric and inguinal lymph nodes, tissue about femur and femur. Except for extensive granular and fatty degenerations, none of these tissues showed anything of importance except the inguinal lymph nodes, diseased femur and tissue adjacent to the diseased bone.

The normal bone marrow was replaced by a dense growth of epitheloid cells, having large densely staining nuclei. These cells were arranged for the most part in irregular solid columns, surrounded by and separated from each other by well marked capillaries. In some portions of the field, dilated capillaries filled with tumor cells were seen. All that remained of the original marrow structure were the bony trabeculæ traversing the tumor mass. No giant cells were seen.

The gelatinous tissue immediately adjacent to the diseased bone consisted of disorganised muscle fibers, in the midst of which were small masses of tumor tissue closely resembling adenocarcinoma. Some areas showed a structure similar to that in the sections of bone, but most of the nests were composed of distinct alveoli, some of which had a single layer of cells, others having three or four layers. There were all gradations from tubules to solid cords of cells. No limiting membrane was discernible, and the muscular tissue was irregularly invaded, tumor cells occurring singly and in small groups between fragmented muscle fibers.

The inguinal lymph nodes were filled with similar tissue having however only a slight tendency to form alveoli. Traversing this tissue were bands of large spindle cells, forming septa between lobules of the round cells.

My time will not permit me to discuss differential diagnosis. Tuberculosis, syphilis, osteomyelitis, benign tumors, periostitis, and sarcoma must all be excluded, with the exception of the last—sarcoma. Clinically, this neoplasm cannot be differentiated from endothelioma, the only point of difference being the age incidence. Primary sarcoma of bone occurs before the age of 40 in from 85 per cent. to 95 per cent. of cases, according to various authors, while endothelioma occurs in about 75 per cent. of cases after the age of 40.

In order that this condition may be recognised before it has advanced beyond the operable stage, I would emphasise certain conclusions whose importance is demonstrated by the case just described and by those previously recorded. These conclusions apply also to the sarcomata.

1. Malignant changes in the bone marrow or in the periosteum may result immediately from trauma, either slight or severe.

2. Pain is present early if the neoplasm originates centrally, *i. e.*, in the marrow, or if it begins in the periosteum and early involves a nerve trunk. On the other hand, pain may be entirely lacking and the first symptoms may be spontaneous fracture.

3. There may or may not be any palpable tumor formation.

4. Malignant disease of the bone marrow is likely to cause a pronounced eosinophilia and may cause a pernicious type of anemia.

5. The long bones are the ones most often primarily affected. Metastases occur in a majority of cases, their seat being most often in other bones. The next most common sites are the lungs, liver and lymphatic glands, in the order named.

In conclusion, let me urge that in any case of injury to bone followed by an undue amount of pain, in all cases of unexplained delayed union of fractures, in all cases of palpable tumor of bone, in all cases of spontaneous fracture, a blood analysis should be made. Eosinophilia, with or without lymphocytosis, anemia or the presence of nucleated red cells, points to serious bone marrow trouble. The urine should be examined for Bence Jones proteid, the presence of which indicates myeloma, and hence contraindicates operative procedure.

